# Impact of Various Mouthwashes on the Color Stability of Hybrid Ceramic and Reinforced Composite CAD/CAM Restorative Materials: An In Vitro Study

**DOI:** 10.3390/ma19040758

**Published:** 2026-02-15

**Authors:** Raul Rotar, Adrian Cândea, Alexandra Măroiu, Andrei-Bogdan Faur, Cristiana Cuzic, Roxana-Elena Rotar, Anca Jivănescu

**Affiliations:** 1 Department of Prosthodontics, University of Medicine and Pharmacy “Victor Babes”, B-dul Revolutiei 1989, No. 9, 300580 Timisoara, Romania; maroiu.alexandra@umft.ro (A.M.); andrei.faur@umft.ro (A.-B.F.); pricop.cristiana@umft.ro (C.C.); jivanescu.anca@umft.ro (A.J.); 2 TADERP Research Center, Department of Prosthodontics, University of Medicine and Pharmacy “Victor Babes”, B-dul Revolutiei 1989, No. 9, 300580 Timisoara, Romania; 3 Department of Endodontics, University of Medicine and Pharmacy “Victor Babes”, B-dul Revolutiei 1989, No. 9, 300580 Timisoara, Romania; roxanaelenabilgar@yahoo.ro

**Keywords:** dental materials, color, ceramics, material testing, mouthwashes

## Abstract

Caries control is essential for the long-term success of dental restorations, and the frequent use of mouthwashes may influence the esthetic properties of restorative materials. This study aimed to evaluate the effect of several commercially available mouthwashes on the color stability of resin-based CAD/CAM restorative materials. Three CAD/CAM materials were investigated: a hybrid ceramic (Cerasmart), a polymer-infiltrated ceramic (Vita Enamic), and a reinforced composite (Brilliant Crios). Rectangular specimens were immersed in Eludril Classic, Listerine Total Care, Listerine Advanced White, and distilled water (control). Color measurements were performed at baseline and after immersion periods up to 24 h using a digital spectrophotometer, and color differences (ΔE_00_) were calculated. Statistical analysis revealed that both mouthwash type and immersion time had a statistically significant effect on color change (*p* ≤ 0.05). All tested materials exhibited ΔE_00_ values exceeding the clinically acceptable threshold. These findings suggest that prolonged mouthwash use may compromise the esthetic longevity of resin-based CAD/CAM restorations. The null hypothesis was that prolonged use of mouthwash does not influence the color of resin-based CAD/CAM materials especially for those containing alcohol.

## 1. Introduction

Controlling caries is essential for the long-term success of dental restorations. Patients with different restorations may benefit from mouth rinsing solutions to help prevent recurrent or secondary caries, particularly around the restoration margins and prepared tooth surfaces [1]. Mouthwashes are commonly used worldwide to address various dental issues, often without the need for a prescription. They serve as adjuncts in the treatment of conditions like gingivitis, periodontitis, halitosis, and in the prevention of caries. Mouthwashes come in a variety of formulations, containing ingredients such as fluoride, antimicrobial agents, preservatives, salt, alcohol, and different flavors. However, frequent use of mouthwash may have adverse effects on both dental restorations and oral tissues [2,3].

Regular use of mouthwashes may elevate the risk of pigmentation, dry mouth, and alterations in the physical properties of composite resin-based restorations [4]. Studies have indicated that alcohol present in mouthrinses may contribute to the softening of resin-composite restorations. However, both alcohol-based and alcohol-free mouthrinses have been shown to impact the hardness of restorative materials [5]. The degree of change in the physical properties of composites, such as microhardness, primarily depends on the type and composition of the materials used [3]. Therefore, any chemical softening resulting from exposure to such products could compromise the clinical longevity of the restorations [6,7].

With the growing emphasis on esthetics, one of the most important success criteria in restorative dentistry is achieving long-term color harmony and stability between the teeth and restorations. Discoloration, whether from external sources or internal factors, can occur even in materials that initially match the color of natural tooth tissue [8].

Chlorhexidine gluconate, a cationic antiseptic and mouthwash, is commonly prescribed by dentists for its bactericidal properties. However, it is also a major concern when it comes to color stability. As a result, it is frequently included in studies focused on the color stability of mouthwashes [9,10,11].

The interaction between CAD/CAM hybrid restorative materials and mouthwashes is largely governed by the chemical composition of the resin matrix and its susceptibility to solvent-induced plasticization and hydrolytic degradation. Resin-based CAD/CAM materials contain methacrylate-based polymer networks that can absorb low-molecular weight solvents, leading to matrix swelling, filler–matrix debonding, and subsequent changes in optical and surface-related properties. Some mouthwashes contain ethanol, essential oils, and acidic components, which are known to act as organic solvents capable of penetrating polymer networks and reducing intermolecular forces within the resin matrix. Other oral rinsing solutions are primarily aqueous but contain chlorhexidine and alcohol, which may promote water sorption and hydrolytic effects within susceptible polymer phases [12].

Although various mouthwashes are available on the market, many have not been examined for their impact on dental restorations. This study aims to assess the effect of several commercially available mouthwashes on the esthetics of composite and hybrid ceramic restorative CAD/CAM materials.

## 2. Materials and Methods

Hybrid ceramic (Cerasmart, GC Corporation, Tokyo, Japan), polymer-infiltrated ceramic (Vita Enamic, Vita Zahnfabrik, Bad Sackingen, Germany), and reinforced composite (Brilliant Crios, Coltene/Whaledent, Langenau, Germany) CAD/CAM materials (Table 1) were subjected to immersion in three types of mouthwashes—Eludril Classic, Listerine Total Care, and Listerine Advanced White (Table 2). Distilled water and coffee were also used for the control group. The coffee solution was prepared by dissolving 0.5 g of instant coffee powder (Davidoff instant coffee, Zino Davidoff Group, Basel, Switzerland) in 50 mL of distilled water. Rectangular samples were cut from the CAD/CAM blocks (A2 Shade) with a thickness of one millimeter. Next step consisted in chairside polishing of each sample following the general recommendations for each material (Table 3).

**Figure 1:**
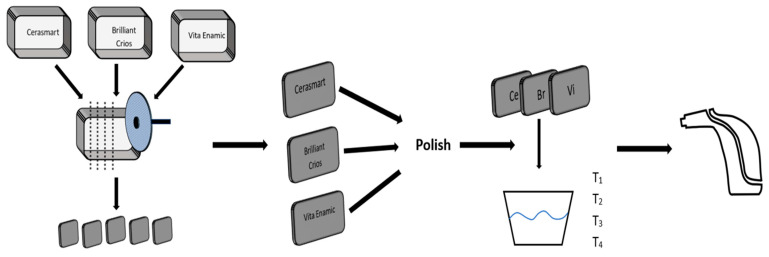
Schematic of the color determination process.

All the samples were washed with distilled water in an ultrasonic bath for five minutes and divided into 4 groups (*n* = 10): Eludril Classic (EC), Listerine Total Care (LTC), Listerine Advanced White (LAW) and distilled water (DW). The samples were stored in containers containing 20 mL mouthwash solutions and placed in an incubator at 37 °C for 1.5 Hours (T_1_), 3 Hours (T_2_), 12 h (T_3_) and 24 h (T_4_). As reference the initial shade of the samples (T0) was determined using a digital spectrophotometer (VITA Easyshade^®^ V, Zahnfabrik GmbH & Co., KG, Bad Säckingen, Germany), which was previously calibrated in accordance with the manufacturer’s recommendations on the calibration block (Figure 1).

The measurement was made in the center of the sample which was hand-held by the operator to prevent any possible influence from external colors due to the translucency of the samples.

The three hours immersion time in mouthwash is similar to one minute of rinsing, twice a day for three months. Therefore, the 24 h immersion time was appreciated to be similar to one minute of rinsing, twice a day for 24 months.

The samples were washed in distilled water, air dried and measured again at the end of each immersion period (T_1_, T_2_, T_3_, T_4_). The color differences between immersion times were determined using the color-difference formula recommended by CIE:

$$\Delta E_{00}=\;\sqrt{{\left(\frac{\Delta L^{\prime }}{k_{L}S_{L}}\right)}^{2}+{\left(\frac{\Delta C^{\prime }}{k_{C}S_{C}}\right)}^{2}+{\left(\frac{\Delta H^{\prime }}{k_{H}S_{H}}\right)}^{2}}$$

where ΔL′: Lightness difference; ΔC′: Chroma difference; ΔH′: Hue difference; $S_{L},S_{C},S_{H}$: Weighting functions; $k_{L},k_{C},k_{H}$: Parametric correction terms (usually all = 1).

Color difference (ΔE_00_) acceptability threshold and interpretation for color adjustment were performed following Table 4.

The CIEDE (1:1:1) system was used, in which the parametric values were taken as ‘1’ [17].

MedCalc (MedCalc Software Ltd., v. 23.4.8-64 bit, Ostend, Belgium) statistical software was used to conduct the statistical analysis by uploading all data. Data distribution was checked for normality using the Kolmogorov–Smirnov test, and the Kruskal–Wallis analysis of variance was conducted to determine statistically significant differences among the restorative materials immersed in each mouthwash.

## 3. Results

**Figure 2:**
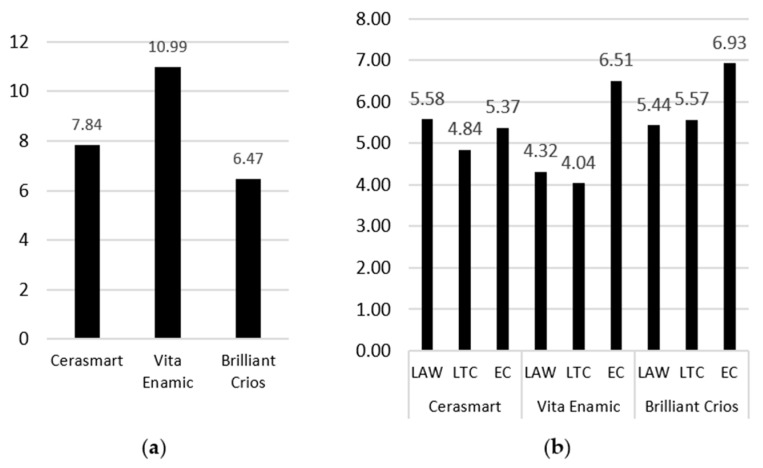
Overall ΔE_00_ values for coffee (**a**) and each material and mouthwash (**b**).

With the *p* value set to ≤0.05, the results of the Kruskal–Wallis test showed that the mouthwash immersion times had a statistically significant effect on the ΔE changes in the three tested mouthwashes: LAW (*p* < 0.006), LTC (*p* < 0.001) and EC (*p* < 0.014). The median and interquartile range (IQR) of ΔE for each immersion time in each type of mouthwash are shown in Table 5. The overall ΔE values for each material and mouthwash are presented in Figure 2.

Statistical analysis revealed significant differences in LAW values among the CAD/CAM groups. As the data did not satisfy the assumptions of normality, a non-parametric Kruskal–Wallis test was applied. The analysis demonstrated a statistically significant difference among the three groups (H = 10.09, df = 2, *p* = 0.0064). Median LAW values varied between groups, with Vita Enamic group presenting the lowest median value (4.32), while Cerasmart and Brilliant Crios groups showed higher and comparable medians (5.58 and 5.44, respectively). Post hoc pairwise comparisons using the Conover test indicated that the Vita Enamic group differed significantly from the Cerasmart group (*p* < 0.05). However, no statistically significant differences were observed between the Vita Enamic group and Brilliant Crios group, nor between the Cerasmart group and Brilliant Crios group (*p* > 0.05). These findings suggest that the mouthwash factor significantly influences ΔE sample values, with the primary difference attributable to the lower values observed in the Vita Enamic group.

Because the data did not meet the assumptions of normality, a non-parametric Kruskal–Wallis test was applied. The test indicated a statistically significant difference in LTC values among the groups (Kruskal–Wallis H = 13.14, df = 2, *p* = 0.0014). Median LTC values differed across groups, with Vita Enamic group showing the lowest median (4.04), followed by Cerasmart group (4.84), and Brilliant Crios group exhibiting the highest median value (5.57). Post hoc pairwise comparisons using the Conover test demonstrated that Vita Enamic group differed significantly from both Cerasmart and Brilliant Crios (*p* < 0.05). In contrast, no statistically significant difference was observed between Cerasmart and Vita Enamic (*p* > 0.05). These results indicate that the mouthwash factor has a significant effect on ΔE sample values, with the Vita Enamic group presenting significantly lower values compared to the other two groups.

Statistical analysis demonstrated significant differences in EC values among the CAD/CAM groups. Given the non-normal distribution of the data, a Kruskal–Wallis test was performed. The analysis revealed a statistically significant difference between the three groups (H = 8.40, df = 2, *p* = 0.0150). Median EC values showed an increasing trend across groups, with the Cerasmart group presenting the lowest median value (5.37), followed by the Vita Enamic group (6.51), while the Brilliant Crios group exhibited the highest median EC value (6.93). Post hoc pairwise comparisons using the Conover test indicated that the Brilliant Crios group differed significantly from the Cerasmart group (*p* < 0.05). No statistically significant differences were detected between the Cerasmart group and Vita Enamic group, or between the Vita Enamic group and Brilliant Crios group (*p* > 0.05). These results suggest that the mouthwash factor significantly affects ΔE sample values, with the most pronounced difference observed between Cerasmart and Brilliant Crios groups.

The coffee immersion was used as a positive control group to compare the magnitude of the staining caused by therapeutic mouthrinses against common dietary extrinsic stainers. The Kruskal–Wallis test revealed a statistically significant difference among the three CAD/CAM materials (H = 46.68, df = 2, *p* < 0.000001). Given the non-parametric nature of the data, a post hoc pairwise comparison was performed using Conover’s test. Post hoc analysis demonstrated statistically significant differences between all pairwise comparisons (*p* < 0.05). Specifically, the Vita Enamic group showed significantly higher values compared to the Cerasmart group and the Brilliant Crios group. Conversely, the Brilliant Crios group exhibited significantly lower values than both the Cerasmart and Vita Enamic groups. The Cerasmart group presented intermediate values but differed significantly from both other groups (Table 6, Figure 2a).

It was observed that in all investigated groups, overall ΔE values were placed above the 3.6 value used as reference meaning that the color was a mismatch (Figure 2b).

ΔC values can be interpreted as follows:

- ΔC > 0 → Increase in chroma. The color becomes more saturated, more vivid, more intense.
- ΔC = 0 → No change in chroma. The saturation remains stable.
- ΔC < 0 → Decrease in chroma. The color becomes less saturated, duller, more grayish.

All examined samples showed values < 0, with the lowest value of −5.07 in the Brilliant Crios group with the LTC mouthwash.

Regarding the ΔH values, they can be interpreted as follows:

- ΔH = 0 → No hue change. The color keeps the same hue.
- ΔH ≠ 0 → Hue shift occurred
- ΔH > 0/ΔH < 0 → indicate the direction of hue rotation in the a*–b* plane (the sign is directional, not qualitative)

The magnitude of ΔH indicates how noticeable the hue shift is; the sign only shows which way the hue moved (Table 7).

## 4. Discussion

Mouthwashes are widely used as adjunctive agents in oral hygiene protocols for the prevention of caries, control of periodontal disease, and management of halitosis. Despite their well-documented benefits for oral health, increasing evidence suggests that prolonged or frequent exposure to mouthwash solutions may adversely affect restorative materials, particularly in terms of color stability and surface properties [5,9,10,11].

The results of the present study demonstrated that all tested CAD/CAM restorative materials exhibited color changes exceeding the clinically acceptable threshold (ΔE_00_ > 3.6) after immersion in the evaluated mouthwashes, regardless of immersion time. These findings indicate that prolonged exposure to commonly used mouthwashes can lead to clinically perceptible and unacceptable discoloration of resin-based CAD/CAM materials with more visible changes for mouthwashes containing alcohol, therefore rejecting the initial hypothesis.

The observed discoloration is consistent with previous reports that identified mouthwashes as potential extrinsic staining agents for composite and hybrid restorative materials [9,10,11,18]. Tanthanuch et al. reported significant color alterations in esthetic restorative materials following exposure to various mouthrinses, attributing these changes to both the chemical composition of the rinses and the resin matrix of the materials [5]. Similarly, El Embaby observed that resin-based restorative materials demonstrated increased discoloration when immersed in mouthwashes containing alcohol and chlorhexidine [10].

In the present study, Eludril Classic (EC) produced the highest overall ΔE values across all materials, followed by Listerine Advanced White (LAW) and Listerine Total Care (LTC). The pronounced discoloration associated with EC may be explained by its high ethanol concentration (~42.8%), chlorhexidine content, and the presence of colorants. Alcohol has been shown to soften the resin matrix by promoting water sorption and polymer degradation, thereby facilitating pigment penetration [2,4,6]. George et al. and Dalmia et al. demonstrated that alcohol-containing mouthrinses significantly reduce the microhardness of resin-based restorative materials, which may indirectly increase susceptibility to staining [18,19].

Chlorhexidine-containing mouthwashes are well known for their staining potential, primarily due to the precipitation of pigmented compounds and interaction with dietary chromogens [9]. The findings of the current study align with previous investigations that consistently reported greater discoloration of restorative materials following exposure to chlorhexidine-based solutions [9,11]. Although chlorhexidine remains the gold standard for antimicrobial efficacy, its impact on restorative esthetics should be carefully considered, particularly in patients with extensive esthetic restorations.

Interestingly, Listerine Advanced White, an alcohol-free mouthwash, also produced clinically unacceptable color changes. This suggests that alcohol is not the sole factor influencing discoloration. Other components such as acidic pH, whitening agents, surfactants, and pyrophosphates may contribute to surface degradation and color instability [3,8]. Yılmaz et al. reported that both alcohol-containing and alcohol-free mouthwashes can adversely affect the surface properties of composite resins, supporting the findings of the present study [3].

Regarding the use of Eludril Classic mouthwash, the manufacturer states that it is indicated as an adjunctive antiseptic treatment in the management of oral and periodontal conditions. It is recommended for short-term use to reduce the oral microbial load and to assist in the control of dental plaque. For administration, Eludril Classic must be diluted prior to use; 10–15 mL of the solution should be diluted with potable water to a final volume of 45 mL. The diluted solution is used to rinse the oral cavity for 30–60 s, twice daily, typically in the morning and evening. Eludril Classic is intended for short-term use under professional supervision, as prolonged use may be associated with adverse effects such as extrinsic tooth staining or transient taste disturbances. The aim of the present study was to investigate whether improper use of this mouthwash could lead to significant color changes in the investigated CAD/CAM restorative materials. Across all investigated mouthwashes, the highest ΔE values were observed in the EC group; however, these differences were not substantially greater than those recorded in the LTC or LAW groups. Even if the spectrophotometer used in this study was calibrated after each measurement, it can still represent a potential source of error. This may account for the ΔE3 value of 1.89 observed in the Vita Enamic group exposed to the LTC mouthwash. The most pronounced color changes were recorded in the Eludril Classic group for the Cerasmart material, particularly at the ΔE3 and ΔE4 evaluation intervals. Based on our clinical observations, a plausible explanation is the presence of a reddish residue, resembling the color of the mouthwash, adherent to the specimen surfaces, which could not be completely eliminated by ultrasonic cleaning.

Cerasmart (GC Corporation, Tokyo, Japan), VITA Enamic (VITA Zahnfabrik Bad Sackingen, Germany) and Brilliant Crios (Coltène/Whaledent, Langenau, Germany) were selected for investigation because they represent distinct categories of contemporary CAD/CAM hybrid restorative materials, combining ceramic and resin components in different structural configurations. Although all three materials are clinically indicated for indirect restorations, they differ fundamentally in microstructure, ceramic–polymer ratio, and network architecture, which are known to influence their mechanical, optical, and interfacial properties. Therefore, the comparative evaluation of these three materials allows for a clinically relevant assessment of how different hybrid material concepts perform under identical experimental conditions, providing insight into material-dependent behavior that cannot be inferred from studies focusing on a single material class. Regarding the restorative materials, Brilliant Crios exhibited the highest ΔE values, followed by Cerasmart and Vita Enamic. This difference may be attributed to variations in resin matrix composition, filler content, and filler–matrix bonding. Resin-based CAD/CAM materials with higher organic matrix content are more prone to water sorption and staining compared to ceramic-dominant materials [13,14,15]. Vita Enamic, a polymer-infiltrated ceramic network material, contains a higher ceramic fraction, which may explain its comparatively lower discoloration than reinforced composite blocks, although the ΔE values still exceeded clinical acceptability.

The immersion protocol used in this study was designed to simulate long-term clinical use, with 24 h of immersion corresponding to approximately two years of twice-daily mouthwash use. This methodology is consistent with previous in vitro studies evaluating the cumulative effects of mouthrinses on restorative materials [3,10,20]. The progressive increase in ΔE values over time observed in the present study further supports the hypothesis that prolonged exposure exacerbates discoloration.

Resin-matrix CAD/CAM materials such as Vita Enamic, Brilliant Crios, and Cerasmart differ in polymer composition, filler type, and matrix–filler integration, which influence their susceptibility to chemical interactions with mouthwash constituents. Mouthwashes contain solvents (e.g., alcohol), surfactants, essential oils, and antimicrobial agents that can penetrate and plasticize the polymer matrix, potentially leading to softening, leaching of unreacted monomers, and breakdown of silane coupling at the filler–matrix interface. In vitro evidence indicates that resin-based CAD/CAM materials exhibit significant color changes when exposed to various mouth rinses, with the extent of discoloration depending on both material composition and mouthwash formulation; for example, alcohol-containing rinses such as Listerine produce greater ΔE_00_ changes in resin nanoceramics including Brilliant Crios compared to other rinses, suggesting enhanced pigment uptake or matrix alteration with prolonged chemical exposure [21,22]. Moreover, polymer-based composites exposed to aggressive solutions show not only discoloration but also measurable reductions in surface microhardness and other mechanical properties, supporting the view that mouthwash constituents can degrade polymer networks beyond surface staining [23]. These chemical effects may facilitate increased water sorption, matrix swelling, micro-crack formation, and eventual weakening of the material structure, which together contribute to both esthetic and functional degradation over time. Therefore, interpreting the observed color change within the context of material degradation mechanisms offers a more comprehensive understanding of how mouthwash chemistries can interact with CAD/CAM restorative materials in the oral environment.

In addition to immersion in distilled water, which served as the negative control, the specimens were also immersed in coffee in order to establish a positive control group for the performed tests. Coffee was selected due to its well-documented high staining potential, attributed to the presence of chromogenic compounds such as polyphenols and tannins, which can readily interact with resin-based materials. Potential chemical affinities exist between coffee constituents and the organic resin matrices of CAD/CAM hybrid materials, particularly through adsorption and absorption mechanisms facilitated by water sorption and matrix plasticization [24,25]. Among the investigated materials, Vita Enamic exhibited the highest degree of pigmentation, which may be explained by its polymer-infiltrated ceramic network structure. This dual-network architecture, combining a porous ceramic scaffold infiltrated with a resin phase, can promote deeper penetration and retention of staining agents compared to the more homogeneous resin composite structures of Cerasmart and Brilliant Crios, thereby resulting in increased discoloration.

A significant limitation of this study is related to the absence of Scanning Electron Microscopy (SEM) or Atomic Force Microscopy (AFM) analyses of the material surfaces after immersion. The interpretation of color stability results should be approached with caution, as the present study did not include a direct assessment of surface topography or material degradation following exposure. In the absence of complementary analyses such as surface profilometry, scanning electron microscopy, or atomic force microscopy, it is not possible to clearly distinguish whether the observed color changes are primarily the consequence of pigment adsorption or are influenced by superficial degradation processes leading to increased surface roughness. Surface roughness is a well-documented factor affecting color perception and staining susceptibility, as microstructural alterations may promote greater retention of chromogenic agents and alter light reflection properties. Therefore, the lack of surface characterization represents a relevant limitation of the study, as it restricts the ability to establish a definitive causal relationship between the experimental exposure and the measured color alterations. Future investigations should incorporate qualitative and quantitative surface analyses to better elucidate the mechanisms underlying color change and to differentiate between extrinsic staining and intrinsic material degradation. Until such data are available, the present findings should be interpreted as indicative rather than conclusive with respect to the mechanisms responsible for color variation.

Another important limitation of the present study is the absence of thermocycling as part of the artificial aging protocol. In the oral environment, restorative materials are subjected to frequent and rapid temperature fluctuations (e.g., ingestion of hot and cold foods or beverages), which induce thermal stresses at the matrix–filler interface, promote hydrothermal degradation, and may contribute to the formation of micro-cracks and increased surface roughness. Such thermal aging has been shown to significantly influence the color stability and surface properties of restorative resins and CAD-CAM materials, with studies reporting detectable changes in color parameters (ΔE/ΔE_00_) and surface roughness after thermal cycling protocols simulating months of clinical service (e.g., 5000–10,000 cycles) in water baths between 5 °C and 55 °C, as well as significant interactions between staining media and thermal cycling on color change outcomes. Recent work highlights that thermocycling affects not only optical properties but also mechanical and surface characteristics that can act synergistically with chemical degradation to accelerate discoloration through enhanced pigment penetration and interfacial breakdown. Without including thermocycling in the experimental design, the potential combined effects of thermal stress and chemical exposure on color change cannot be fully assessed, limiting the extrapolation of in vitro findings to the complex intraoral environment [23]. While the current in vitro setup provides valuable data, the absence of thermal stress simulation limits the clinical relevance regarding the long-term behavior of the tested CAD/CAM materials in the oral environment. 

Other limitations of this study are the small number of restorative materials and mouthwashes that were investigated, as well as the in vitro design, which does not fully replicate the dynamic oral environment, including salivary flow, temperature fluctuations, mechanical wear, and dietary staining challenges. In vitro studies remain essential for isolating the effects of specific chemical agents on restorative materials under standardized conditions. Future research should include surface roughness analysis, gloss evaluation, and in vivo assessments to better understand the clinical implications of long-term mouthwash use on CAD/CAM restorations.

## 5. Conclusions

Within the limitations of this in vitro study, the following conclusions can be drawn:

- All tested CAD/CAM restorative materials exhibited clinically unacceptable color changes after immersion in the evaluated mouthwashes, as ΔE_00_ values consistently exceeded the acceptability threshold (ΔE_00_ > 3.6), regardless of immersion time.
- Mouthwash type significantly influenced color stability, with Eludril Classic producing the greatest discoloration, followed by Listerine Advanced White and Listerine Total Care. The pronounced effect of Eludril Classic may be related to its high alcohol content, chlorhexidine presence, and added colorants.
- Immersion time had a statistically significant effect on color change, indicating that prolonged exposure to mouthwash solutions leads to cumulative and progressive discoloration of resin-based CAD/CAM materials.
- Vita Enamic showed the highest color stability among the tested materials, although still clinically unacceptable after long-term exposure. Therefore, material selection is critical for color preservation.
- Alcohol-free mouthwashes are not necessarily free from discoloration risk, as Listerine Advanced White caused significant color changes, suggesting that factors such as surfactants and other active components also contribute to color instability.
- From a clinical perspective, long-term and frequent use of mouthwashes may compromise the esthetic longevity of resin-based CAD/CAM restorations, particularly in patients with high esthetic demands or extensive restorative work.

Further in vivo studies are recommended to validate these findings under clinical conditions and to investigate additional surface properties that may influence the long-term esthetic performance of CAD/CAM restorative materials.

## Figures and Tables

**Table 1:** Material composition of the CAD CAM blocks [13,14,15].

Materials	Manufacturer	Type	Composition	Filler Ratio
				% by wt.
Cerasmart	GC Corporation, Tokyo, Japan	Hybrid Ceramic CAD/CAM block	Bis-MEPP, UDMA, DMA silicon dioxide (20 nm), barium glass (300 nm),	71% wt.
			nanoparticle-filled resin containing silica monomers	
Vita Enamic	Vita Zahnfabrik, Bad Sackingen, Germany	Polymer-infiltrated CAD/CAM block	Ceramic: silicon dioxide 58–63%, aluminum oxide 20–23%, sodium oxide 9–11%, potassium oxide 4–6%, boron trioxide 0.5–2%, zirconia and calcium oxide.	86% wt.
			Polymer part (25%): UDMA and TEGDMA	
Brilliant Crios	Coltene/Whaledent, Langenau, Germany	Reinforced composite CAD/CAM block	Cross-linked methacrylates (Bis-GMA, BIS-EMA, TEGMA), barium glass (particle size < 1.0 μm) and silica (particle size < 20 μm)	71% wt.

**Table 2:** Mouthwashes’ composition.

Mouthwash	Manufacturer	Composition	pH	Alcohol
Eludril Classic	Pierre Fabre Dermo-Cosmétique Lavaur, France	Chlorhexidine digluconate (0.10%) Chlorobutanol hemihydrate (0.50%), Ethanol (~42.8% *v*/*v*), Glycerol (Glycerin), Purified Water, Aroma (Mint flavor), Menthol (L-menthol), Sodium saccharin, Red dye CI 16255 (Ponceau 4R), Diethylhexyl sodium sulfosuccinate	Not stated (slightly acidic based on composition)	yes
Listerine Total Care	Johnson and Johnson, Skillman, NJ, USA	Sodium Fluoride (0.05% *w*/*v*), Eucalyptol (0.091% *w*/*v*), Thymol (0.063% *w*/*v*), Menthol (0.05% *w*/*v*), Zinc Chloride (0.09% *w*/*v*), Alcohol (21.6% *v*/*v*), Sorbitol, Poloxamer 407, Benzoic Acid, Sodium Benzoate, Sodium Saccharin, Sucralose, Water (Aqua), Colorants (e.g., Red 40, Blue 1), Methyl Salicylate	4.2 to 5.5	yes
Listerine Advanced White	Johnson and Johnson, Skillman, NJ, USA	Aqua (Water), Sorbitol, Propylene Glycol, Tetrapotassium Pyrophosphate, Pentasodium Triphosphate, Citric Acid, Poloxamer 407, Aroma, Sodium Methyl Cocoyl Taurate, Caprylyl Glycol, Eucalyptol, Thymol, Sodium Saccharin, Menthol, Sodium Fluoride, Sucralose	Not stated (slightly acidic based on composition)	no

**Table 3:** Chairside polishing sequence.

Material	
Cerasmart Brilliant Crios Vita Enamic	Silicone polisher—preliminary polishing 6000 rpm
	Bison brush—polishing 6000–10,000 rpm
	Goat hair-brush—polishing 6000–10,000 rpm
	Cotton buff + Renfert polish all in one paste—high luster polishing—10,000 rpm
	(Renfert all in one starter kit—Renfert GmbH, Hilzingen, Germany)

**Table 4:** Color difference acceptability threshold [16].

ΔE_00_ Threshold	Interpretation
≤0.8	Excellent match
>0.8 ≤ 1.8	Acceptable match
>1.8 ≤ 3.6	Mismatch (moderately unacceptable)
>3.6 ≤ 5.4	Mismatch (clearly unacceptable)
>5.4	Mismatch (extremely unacceptable)

**Table 5:** Median (IQR) of ΔE for each mouthwash immersion time.

Material	Mouthwash	ΔE_1_	ΔE_2_	ΔE_3_	ΔE_4_
Cerasmart	LAW	5.54 (0.38)	5.60 (1.35)	5.72 (2.61)	5.66 (2.37)
	LTC	4.16 (2.81)	5.01 (2.20)	4.58 (1.55)	6.15 (2.93)
	EC	5.77 (2.35)	5.49 (1.63)	6.78 (0.4)	3.99 (0.81)
Vita Enamic	LAW	3.92 (2.65)	5.67 (2.87)	4.12 (2.19)	5.59 (2.70)
	LTC	4.16 (3.52)	4.57 (3.19)	1.89 (0.93)	5.12 (1.07)
	EC	6.19 (1.18)	6.93 (1.31)	4.42 (0.23)	7.15 (0.70)
Brilliant Crios	LAW	5.39 (0.82)	5.55 (0.76)	5.54 (1.32)	5.22 (1.06)
	LTC	5.26 (4.84)	5.16 (0.92)	6.11 (0.72)	8.34 (1.03)
	EC	9.22 (0.36)	9.06 (0.86)	4.64 (0.27)	5.48 (0.25)

**Table 6:** Median (IQR) of ΔE for each coffee immersion time.

Material	ΔE_1_	ΔE_2_	ΔE_3_	ΔE_4_
Cerasmart	5.90 (4.59)	7.57 (2.68)	9.19 (3.66)	8.13 (2.54)
Vita Enamic	7.48 (3.72)	10.17 (4.68)	11.39 (4.64)	12.22 (4.13)
Brilliant Crios	5.51 (0.93)	7.65 (2.09)	6.41 (0.48)	7.07 (1.52)

**Table 7:** Overall ΔC and ΔH values for each material and mouthwash.

Material	Mouthwash	ΔC_1_	ΔC_2_	ΔC_3_	ΔC_4_
Cerasmart	LAW	−3.2 (0.20)	−3.0 (0.88)	−3.2 (1.43)	−3.4 (1.38)
	LTC	−2.8 (1.70)	−3.55 (1.48)	0.5 (0.40)	−3.5 (1.40)
	EC	−3.8 (1.20)	−4.05 (0.90)	5.4 (0.37)	0.4 (0.60)
Vita Enamic	LAW	−3.3 (2.35)	−4 (1.63)	−3.0 (1.10)	−4.05 (1.70)
	LTC	−3.2 (2.33)	−3.45 (2.03)	0.2 (0.08)	−3.4 (2.33)
	EC	−4.8 (2.40)	−5.4 (2.68)	1.8 (2.23)	4.9 (1.05)
Brilliant Crios	LAW	−3.35 (0.60)	−3.1 (0.63)	−3.5 (0.85)	−3.85 (1.00)
	LTC	−4.5 (4.90)	−4.6 (0.58)	−6.2 (0.80)	−8.2 (1.10)
	EC	−9.2 (0.43)	−8.95 (0.82)	−2.6 (0.75)	−0.55 (1.25)
**Material**	**Mouthwash**	**ΔH_1_**	**ΔH_2_**	**ΔH_3_**	**ΔH_4_**
Cerasmart	LAW	−8.8 (2.77)	−7.55 (0.10)	−6.25 (4.52)	−6.2 (3.12)
	LTC	−3.45 (6.27)	−8.7 (3.57)	7.6 (1.27)	−7.8 (4.15)
	EC	−2.0 (3.62)	−8.2 (3.67)	15.75 (0.50)	12.95 (2.70)
Vita Enamic	LAW	−3.0 (5.45)	−4.7 (1.35)	−3.0 (3.80)	−5.05 (3.90)
	LTC	−1.75 (3.50)	−3.35 (4.07)	6.4 (1.80)	−1.2 (3.65)
	EC	0.2 (4.55)	−4.6 (4.27)	9.3 (3.92)	10.1 (2.55)
Brilliant Crios	LAW	−5.55 (4.85)	−4.65 (4.47)	−4.5 (3.67)	−5.25 (2.85)
	LTC	−3.9 (11.17)	−4.3 (1.65)	−3.05 (0.30)	−10.2 (2.20)
	EC	−5.9 (0.47)	−9.55 (1.55)	2.35 (0.70)	3.65 (1.50)

## Data Availability

The raw data supporting the conclusions of this article will be made available by the authors on request.

## Abbreviations

The following abbreviations are used in this manuscript:

CAD/CAM	Computer aided design/Computer aided manufacturing
EC	Eludril Classic
LTC	Listerine Total Care
LAW	Listerine Advanced White
DA	Distilled water
